# Association between Novel Object Recognition/Spontaneous Alternation Behavior and Emission of Ultrasonic Vocalizations in Rats: Possible Relevance to the Study of Memory

**DOI:** 10.3390/brainsci11081053

**Published:** 2021-08-09

**Authors:** Giulia Costa, Marcello Serra, Nicola Simola

**Affiliations:** Department of Biomedical Sciences, University of Cagliari, 09042 Monserrato, Italy; gcosta@unica.it (G.C.); marcelloserra@unica.it (M.S.)

**Keywords:** 22-kHz calls, 50-kHz calls, affect, aversion, NOR, reward, working memory, Y maze

## Abstract

Rats emit ultrasonic vocalizations (USVs) in situations with emotional valence, and USVs have also been proposed as a marker for memories conditioned to those situations. This study investigated whether USV emissions can predict and/or be associated with the behavior of rats in tests that evaluate unconditioned memory. To this end, rats were subjected to “tickling”, a procedure of heterospecific play that has emotional valence and elicits the emission of USVs, and afterwards evaluated in the novel object recognition test (NOR) and in the single trial continuous spontaneous alternation behavior (SAB) test in a Y maze. The number of 22-kHz USVs (aversive) and 50-kHz USVs (appetitive) emitted in response to tickling and during NOR and SAB tests were scored, and the correlations among them and with rats’ behavior evaluated. Rats emitted 50-kHz USVs, but not 22-kHz USVs, during the NOR and SAB tests, and such calling behavior was not linked with the behavioral readouts indicative of memory function in either test. However, rats that prevalently emitted 22-kHz USVs in response to tickling displayed an impaired NOR performance. These findings suggest that measuring the emission of USVs could be of interest in studies of unconditioned memory, at least with regard to 22-kHz USVs.

## 1. Introduction

Rats emit ultrasonic vocalizations (USVs) in response to and/or anticipation of various stimuli that possess emotional valence [1,2,3]. Two families of USVs have been characterized in rats, which possess different acoustic features and carry different information about the emotional valence of stimuli and situations [4]. Situations that possess negative emotional valence for rats (i.e., encounters with or detection of aggressive conspecifics, predators or unfamiliar humans) elicit the emission of the so-called 22-kHz USVs, which have a long duration (longer than 300 ms, although 22-kHz of short duration have also been described), sound frequency between 20–35 kHz, and scarce frequency modulation [4]. Conversely, situations that possess positive emotional valence for rats (i.e., non-aggressive interactions with conspecifics or familiar humans and administration of drugs with rewarding properties) elicit the emission of the so-called 50-kHz USVs, which have a short duration (generally 30–40 ms), sound frequency between 35–80 kHz (which can be even higher), and may possess marked frequency modulation [4]. On these bases, measuring the emission of USVs is increasingly emerging as a useful marker in rat studies that evaluate emotional behavior and the factors that may modify the emotional state, both in physiological conditions and experimental models of brain disease [5,6].

Of particular interest in USV research is the evidence that rats may call in anticipation of stimuli that possess emotional valence. Thus, rats may emit either anticipatory 22-kHz USVs when re-exposed to an environment where they received aversive electric foot-shocks [7,8], or anticipatory 50-kHz USVs in response to environmental cues previously associated with pharmacological or non-pharmacological rewards [9,10,11,12,13,14,15,16,17]. Taken together, these findings have suggested that calling behavior may be a marker of conditioned memories for stimuli/situations that possess emotional valence. Notably, a very recent study has confirmed and extended this hypothesis, by demonstrating that rats tested in a fear conditioning paradigm with electric shocks of low intensity displayed minimal freezing behavior, but showed a decreased emission of frequency-modulated (FM) 50-kHz USVs that persisted even after the extinction of freezing behavior [18]. Elucidating the relationship between the emission of USVs and memory in rats is of interest, since experimental evidence exists to suggest the presence of an interplay between modifications in the emotional state and changes in memory function [19,20]. Moreover, measuring USVs could potentially complement the classical behavioral markers that are evaluated in rat studies of memory (i.e., preference for items, patterns of arm exploration in mazes), thus increasing the quantity of information that can be collected in those studies.

The present study was performed to elucidate whether the emission of USVs can be predictive of and/or associated with the behavior of rats in tests that are used to evaluate memory and do not employ conditioned stimuli and primary reinforcers, but rely on the animals’ preference for novelty [21,22]. To this end, rats were first subjected to “tickling”, a procedure that consists of the interaction with the hands of a familiar human that resembles “rough and tumble” play of juvenile rats, and which may be associated with changes in the emotional state and emission of USVs [23]. Afterwards, rats were evaluated in the novel object recognition test (NOR) as well as in the single trial continuous spontaneous alternation behavior (SAB) test in a Y maze. The number of 22-kHz USVs (aversive) and 50-kHz USVs (appetitive) emitted in response to tickling as well as during NOR and SAB tests were scored, and the existence of a relationship between the magnitude/type of calling behavior and the performance of rats in each of the memory tests carried out (i.e., preference for novel objects, SAB) was investigated.

## 2. Materials and Methods

### 2.1. Subjects

A total of 40 male Sprague–Dawley rats (Envigo, Italy) were involved in the study. Rats weighed 100–125 g (aged 28–35 days) at the beginning of the experiments and were housed 5 animals per cage in standard polycarbonate cages (L 48 cm × H 21 cm × W 38 cm) under a 12-h light/dark cycle (lights on at 08:00 h). Rats had free access to standard laboratory chow and water, except during the experiments that were performed between 10:00 and 16:00 h. The study was carried out in accordance with the guidelines for animal experimentation of the EU directives (2010/63/EU, L.276; 22/09/2010), and with the guidelines issued by the Committee for Animal Welfare (OPBA) of the University of Cagliari. All the appropriate procedures were followed to minimize animal discomfort and number of animals used.

### 2.2. Experimental Plan

**Figure 1:**
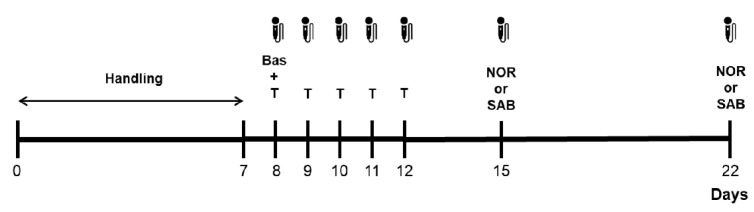
Experimental plan. A total of 40 rats were gently handled daily for 7 consecutive days. The day after, rats were evaluated a single time for their basal calling behavior and, starting the same day, subjected to tickling for 5 consecutive days. On days 15 and 22, rats were arranged in two groups of 20 subjects and tested once either in the novel object recognition test or in the single trial continuous spontaneous alternation behavior test in a Y maze. Testing was done in a counterbalanced manner and the emission of ultrasonic vocalizations was recorded throughout the experiments. Bas = recording of basal calling behavior before the beginning of tickling; NOR = novel object recognition test; SAB = spontaneous alternation behavior; T = tickling.

All experiments were performed in a quiet room under an illumination of 40 lx and the experimental plan is described in Figure 1. Rats were gently handled daily (5 min) for 7 consecutive days; thereafter, they were evaluated a single time for their basal emission of USVs (day 8). Afterwards, starting immediately after the evaluation of basal calling behavior, all rats were subjected to tickling for 5 consecutive days. Finally, starting 3 days after the completion of tickling, all rats were evaluated in the NOR test and in the single trial continuous SAB test in a Y maze. Evaluations were performed in a counterbalanced manner by dividing the rats in two groups of 20 subjects: one group was evaluated first in the NOR test and then in the SAB test, while the opposite was done in the other group. Evaluations were separated by a 7-day interval and the emission of USVs was recorded throughout the experiments.

### 2.3. Evaluation of Basal Calling Behavior

Rats were individually placed in medium-sized polycarbonate cages (L 42 cm × H 14 cm × W 14 cm) without bedding and left to explore the environment freely for 5 min, during which the emission of USVs was recorded.

### 2.4. Tickling

Beginning immediately after the evaluation of basal calling behavior, tickling was performed in medium-sized polycarbonate cages (L 42 cm × H 14 cm × W 14 cm) without bedding, according to the procedure previously described [23,24]. Briefly, tickling was done with one hand and consisted of scaled-down and rapid movements of fingers and hand (i.e., “belly tickle”, “flip over”, “hand chase”, “neck tickle”, and “push and drill”). Each rat was subjected to 1 cycle of tickling × day × 5 consecutive days, and each cycle of tickling consisted of blocks of 15 s of baseline with no tactile stimulation, followed by blocks of 15 s of tactile stimulation, repeated for a total of 2 min [23]. The experimenter’s hand was kept still inside the cage during the baseline blocks.

### 2.5. Novel Object Recognition Test

The NOR test is a behavioral paradigm that allows to evaluate non-spatial working memory in rodents in the absence of emotional and learning components [21]. NOR tests were performed in polycarbonate cages (L 42 cm × W 14 cm × H 30 cm) that were enclosed by cardboard walls (H 50 cm) and had their bottom covered with an elevated grid (3 cm) having a handful of sawdust below it [25]. The objects to be discriminated in the NOR test were plastic-made, had different shape and color, and were devoid of genuine significance and emotional valence for rats. The experimental procedure consisted of three sessions: habituation (S0), acquisition (S1), and testing (S2), and rats were individually evaluated in each of these sessions. For habituation, each rat was placed in the test cage in the absence of objects and left to explore the environment freely for a single trial (5 min). Twenty-four hours after S0, acquisition (S1) was performed by placing each rat in the test cage together with two copies of an object (familiar objects); the rat was allowed to freely explore the objects for 3 min. The testing session (S2) was performed 60 min after S1 and consisted of the exposure of each rat for 3 min to a pair of objects made of one copy of the objects already encountered in S1 and of an object that the rat had never encountered before (novel object). Objects were always placed in the vicinity of the two adjacent corners along the long side of the cage, leaving a distance between the objects and between the objects and the walls of the cage that allowed the rats to turn around the objects. The exploration times and the inter-session times were selected based on previous studies of our group, demonstrating that such times are adequate to reveal the presence of memory impairment in rodents that are evaluated in the NOR test [25,26,27]. Object exploration was scored when a rat sniffed, bit, or touched a specific object, whereas object exploration was not scored when rats circled around the objects and/or sat on them. The objects were cleaned at the end of each session to take away olfactory traces and counterbalanced for location (right or left side of the cage) and status (old or novel). The behavior of rats was videotaped during S1 and S2, and later evaluated to determine the following parameters: (a) seconds spent in object exploration during S1 and S2; (b) percentage of time spent exploring the novel and old objects during S2.

### 2.6. Single-Trial Continuous Spontaneous Alternation Behavior Test in a Y Maze

Evaluation of continuous SAB in a Y maze is an experimental paradigm that allows to assess the sensory/attentional functions and spatial working memory in rodents, and does not rely on conditioned stimuli and primary reinforcers [22]. The Y maze used was made of black PVC and had three equally sized symmetrical arms (L 50 cm × W 20 cm × H 35 cm) that converged onto a central triangular area; moreover, the maze had its bottom covered with sawdust. For testing, each rat was individually placed in the central triangular area and left to explore freely the entire maze for a single 8 min trial. In order to remove olfactory cues, the sawdust was changed and the maze was cleaned in between each rat. The rats’ performance was videotaped to score for: (i) number of arm entries; (ii) sequence of arm entries, to calculate spontaneous alternation. A rat was considered inside an arm when it had all its four paws inside a specific arm, and spontaneous alternation was defined as successive entries into all the three arms of the maze in overlapping triplet sets, and expressed as the percentage of actual to possible alternations (defined as the total number of entries in arms − 2) × 100 [26].

### 2.7. Recording of Ultrasonic Vocalizations

The emission of USVs was recorded in each step of the experimental protocol by means of ultrasonic microphones (CM16/CMPA, Avisoft, Berlin, Germany) that were connected to an ultrasound recording device (Ultrasound Gate 116 Hb, Avisoft, Berlin, Germany). Intensity gain was kept at a constant level throughout recordings. For recording in cages (i.e., basal emission of USVs, tickling, NOR test), a single microphone was hung on a support and centered above the cage at a distance of 40 cm from the bottom. For recording in the Y maze, three microphones were used; each microphone was hung sideways from the center of the right wall of each arm of the maze at a distance of 30 cm from the bottom. The duration of USV recordings matched that of behavioral evaluation in each step of the experimental protocol. 

### 2.8. Data Collection and Statistical Analyses

**Figure 2:**
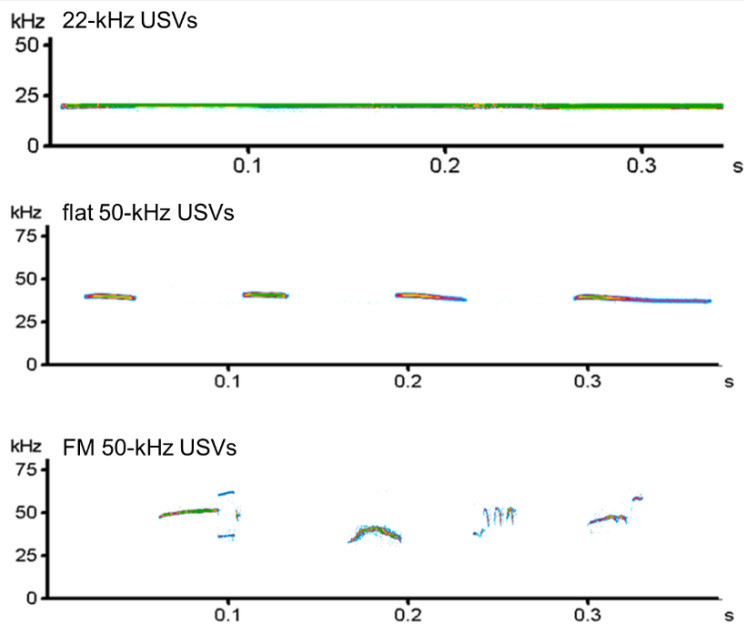
Example of spectrograms of 22-kHz and 50-kHz ultrasonic vocalizations recorded in the present study. The vocalizations reported in the figure are independent calls emitted by different rats. FM = frequency-modulated; USVs = ultrasonic vocalizations.

USV recordings were converted into spectrograms by means of the software SASLab Pro 4.52 (Avisoft, Berlin, Germany), which was also used to count the number of 22-kHz and 50-kHz USVs isolated in each spectrogram and their acoustic parameters (duration, maximum frequency, minimum frequency) [28]. In addition, 50-kHz calls were categorized into flat and FM according to the criteria proposed by Wright and coworkers [29]. Examples of USVs recorded in the present study are provided in Figure 2.

Means ± S.E.M. were calculated for the following parameters: (i) number and acoustic parameters of 22-kHz and 50-kHz USVs (total and categorized, when appropriate) emitted at each step of the experimental protocol; (ii) percentages of time (seconds) spent exploring the objects in S1 and S2 of the NOR test; (iii) percentages of SAB and number of entries in the arms of the Y maze. USV data were analyzed by an experimenter blind to the conditions of recording. All data obtained in the present study were tested for normality with the Kolmogorov–Smirnov test and analyzed accordingly with one of the following tests: (i) one-way or two-way analysis of variance (ANOVA) followed by Bonferroni’s multiple comparisons test, when appropriate; (ii) Kruskal–Wallis test followed by Dunn’s multiple comparisons test, when appropriate; (iii) Student’s t-test; (iv) Mann–Whitney U test or Wilcoxon test. Moreover, and when appropriate, Spearman’s test was used to correlate the behavioral readouts obtained in the NOR and SAB tests (i.e., preference for novel objects, SAB, number of arm entries) with the number of 22-kHz and 50-kHz USVs emitted either in response to tickling or during memory testing. Bonferroni correction for multiple comparisons was applied to correlation analysis, when appropriate. Finally, in order to further clarify whether the emission of USVs in response to tickling could predict the behavior of rats in the NOR and SAB tests, an additional analysis was performed by dividing the rats in three groups according to the prevalent type of calls emitted in response to tickling, calculated as the total number of calls emitted over the five sessions of tickling. Subdivision of rats was performed as follows: (i) rats that emitted only 50-kHz calls, (ii) rats that emitted more 50-kHz calls than 22-kHz calls, (iii) rats that emitted more 22-kHz calls than 50-kHz calls. Statistical analysis was performed with Prism 8 (GraphPad, San Diego, CA, USA) for Windows. Significance was set at *p* < 0.05 for each analysis. Two rats were excluded from the analysis of USVs emitted during Y maze exploration due to a loss of spectrograms. The acoustic parameters of the USVs recorded in the present study were in the range of those previously reported for 22-kHz and 50-kHz calls [4] (data not shown).

## 3. Results

### 3.1. Basal Levels of Calling Behavior

Rats emitted a very low number of 50-kHz USVs when exposed to a novel cage in the absence of bedding (average number of calls = 0.23 ± 0.05 in 5 min of recording), and no emission of 22-kHz USVs was observed in the same situation.

### 3.2. Emission of Ultrasonic Vocalizations in Response to Tickling

**Figure 3:**
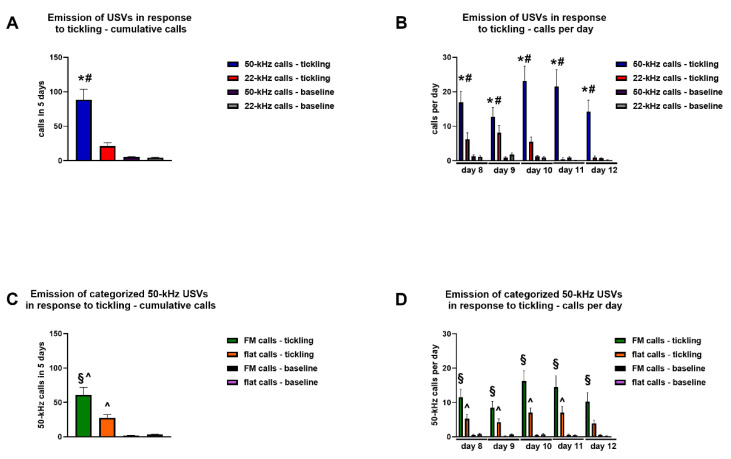
Emission of ultrasonic vocalizations in response to tickling. Rats underwent 1 cycle of tickling × day × 5 consecutive days, and each cycle of tickling consisted in blocks where no tactile stimulation was performed (baseline) alternated with blocks where tactile stimulation was performed (tickling). The emission of ultrasonic vocalizations was recorded throughout each cycle of tickling performed. Panel (**A**) demonstrates the cumulative numbers of 22-kHz and 50-kHz calls emitted over the 5 sessions of tickling. Panel (**B**) demonstrates the numbers of 22-kHz and 50-kHz calls emitted in each session of tickling. Panel (**C**) demonstrates the cumulative numbers of categorized 50-kHz calls emitted over the 5 sessions of tickling. Panel (**D**) demonstrates the numbers of categorized 50-kHz calls emitted in each session of tickling. * indicates a significant difference vs. 50-kHz calls—baseline. # indicates a significant difference vs. 22-kHz calls—tickling. § indicates a significant difference vs. FM calls—baseline. ^ indicates a significant difference vs. flat calls—baseline. FM = frequency modulated; USVs = ultrasonic vocalizations. *n* = 40.

Tickling significantly stimulated the emission of USVs in rats. The Kruskal–Wallis test showed the presence of significant modifications in the cumulative number of calls emitted over the 5 days of tickling (K = 53.22, *p* < 0.01), and Dunn’s test for multiple comparisons revealed that a significant increase in the emission of 50-kHz USVs (*p* < 0.01), but not 22-kHz USVs, occurred during tickling sessions, compared with baseline sessions when the hand of the experimenter was passively left inside the cage (Figure 3A). Moreover, the Kruskal–Wallis test showed the presence of significant changes in the number of calls emitted in each day of tickling (day 8, K = 46.47, *p* < 0.01; day 9, K = 27.63, *p* < 0.01; day 10, K = 46.90, *p* < 0.01; day 11, K = 82.90, *p* < 0.01; day 12, K = 57.86, *p* < 0.01), and Dunn’s test for multiple comparisons revealed that a significant increase in the emission of 50-kHz USVs (days 8–12, *p* < 0.01), but not 22-kHz USVs, occurred during tickling sessions, compared with baseline sessions (Figure 3B).

The Kruskal–Wallis test showed the presence of significant modifications in the cumulative number of categorized 50-kHz USVs emitted over the 5 days of tickling (K = 71.43, *p* < 0.01), and Dunn’s test for multiple comparisons revealed that a significant increase in the emission of FM (*p* < 0.01) and flat (*p* < 0.01) calls occurred during tickling sessions, compared with baseline sessions when the hand of the experimenter was passively left inside the cage (Figure 3C). Moreover, the Kruskal–Wallis test showed the presence of significant changes in the number of categorized 50-kHz USVs emitted on each day of tickling (day 8, K = 61.24, *p* < 0.01; day 9, K = 45.80, *p* < 0.01; day 10, K = 57.45, *p* < 0.01; day 11, K = 43.80, *p* < 0.01; day 12, K = 40.24, *p* < 0.01). Dunn’s test for multiple comparisons revealed that a significant increase occurred during tickling sessions for the emission of FM calls in days 8–12 of tickling (*p* < 0.01 for all days), and for the emission of flat calls in days 8–11 of tickling (days 8, 10, and 11, when the hand of the experimenter was passively left inside the cage, *p* < 0.01; day 9, *p* < 0.05), all compared with baseline sessions (Figure 3D).

### 3.3. Novel Object Recognition Test and Emission of Ultrasonic Vocalizations

**Figure 4:**
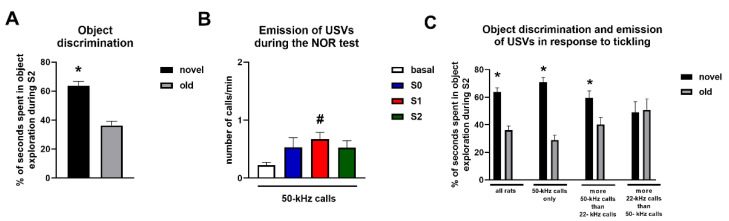
Object discrimination and emission of ultrasonic vocalizations during the novel object recognition test. Panel (**A**) reports the percentage of time spent exploring the novel and old objects in all the rats tested. Panel (**B**) demonstrates the emission of 50-kHz ultrasonic vocalizations in the different sessions of the novel object recognition test. Panel (**C**) reports the percentages of time spent exploring the novel and old objects when rats were grouped according to the prevalent type of ultrasonic vocalizations (i.e., 22-kHz or 50-kHz calls) emitted in response to tickling. * Indicates a significant difference vs. old objects. # Indicates a significant difference vs. basal. NOR = novel object recognition; S0, S1, and S2 = session 0, 1, and 2 of the novel object recognition test. *n* = 40 for panels A and B and for the group “all rats” in panel (**C**); *n* = 21 for the group “50-kHz calls only” in panel (**C**); *n* = 12 for the group “more 50-kHz calls than 22-kHz calls” in panel (**C**); *n* = 7 for the group “more 22-kHz calls than 50-kHz calls” in panel (**C**).

Within-group analysis performed in all rats revealed the presence of a preference for novel objects (Figure 4A), since during S2, rats spent a significantly higher percentage of time exploring the novel objects than the old objects (paired *t*-test, t = 4.67, df = 39, *p* < 0.01). Counterbalancing of NOR and Y maze testing did not influence the preference for novel objects in the NOR test. Indeed, the percentage of time spent in novel object exploration was comparable between rats that were first evaluated in the NOR test and then in the Y maze (65.29 ± 4.33) and rats that were first evaluated in the Y maze and then in the NOR test (62.81 ± 4.01).

During the different phases of the NOR test, rats displayed a very scarce emission of 50-kHz USVs (S0, average number of calls = 2.68 ± 0.8 in 5 min of recording; S1, average number of calls = 2.03 ± 0.35 in 3 min of recording; S2, average number of calls = 1.58 ± 0.36 in 3 min of recording) and no emission of 22-kHz USVs was observed. Nevertheless, the Kruskal–Wallis test revealed that the number of 50-kHz USVs emitted per minute during the NOR test was significantly higher compared with that recorded during the evaluation of basal calling behavior performed before the beginning of tickling (K = 12.03; *p* < 0.05), and Dunn’s test for multiple comparisons revealed that this effect occurred during S1 (*p* < 0.05) (Figure 4B).

Differences in object discrimination were observed when rats were divided in three groups based on the prevalent type of USVs (i.e., 22-kHz or 50-kHz calls) that were emitted in response to tickling. Within-group analysis revealed a significant increase in the percentage of time spent exploring the novel objects during S2 in the group of rats that emitted only 50-kHz USVs in response to tickling (paired *t*-test, t = 6.17, df = 20, *p* < 0.01), as well as in the group of rats that emitted more 50-kHz calls than 22-kHz calls in response to tickling (Wilcoxon test, W = 12, *p* < 0.05). Conversely, within-group analysis revealed that rats that emitted more 22-kHz calls than 50-kHz calls in response to tickling spent comparable percentages of time exploring the novel and old objects during S2 (paired t-test, t = 0.12, df = 6, *p* = 0.91) (Figure 4C).

The results of the within-group analysis were confirmed by the between-group analysis with two-way ANOVA, which revealed a significant effect of object (F1,152 = 41.32, *p* < 0.001) and a significant interaction object × group (F3,152 = 5.49, *p* = <0.01), but no significant effect of group (F3,152 = 0.01, *p* = 0.99). Bonferroni’s multiple comparisons test revealed that a significant discrimination between novel and old objects occurred in all rats (*p* < 0.01), in the group of rats that emitted only 50-kHz USVs in response to tickling (*p* < 0.01), as well as in the group of rats that emitted more 50-kHz calls than 22-kHz calls in response to tickling (*p* = 0.036), but not in the group of rats that emitted more 22-kHz calls than 50-kHz calls in response to tickling (*p* = 0.99) (Figure 4C). Bonferroni’s multiple comparisons test revealed no significant group differences in the percentages of time spent in novel object exploration (Figure 4C).

No group differences in the cumulative times of object exploration during S1 and S2 were observed when rats were grouped according to the prevalent type of USVs emitted in response to tickling (Table 1).

**Figure 5:**
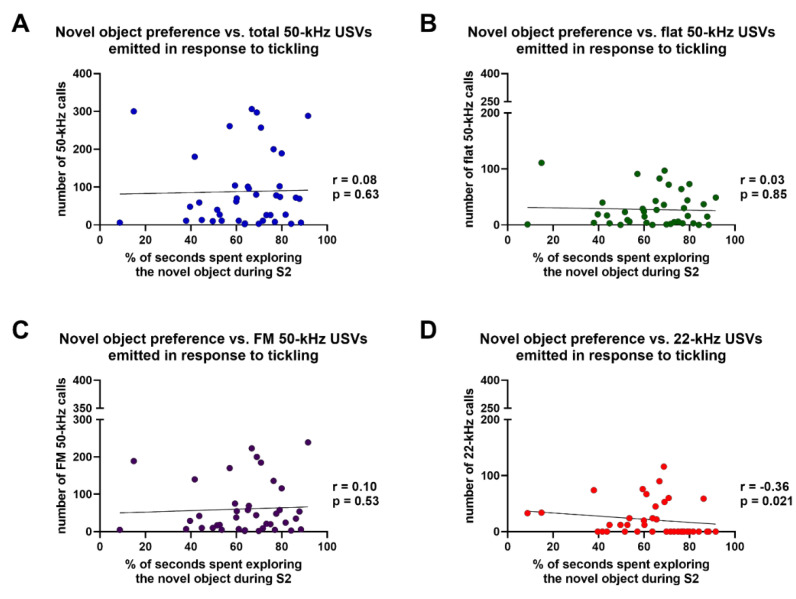
Results of Spearman’s correlation test of the percentages of time spent exploring the novel objects during S2 of the novel object recognition test and the number of total 50-kHz ultrasonic vocalizations (**A**), flat 50-kHz ultrasonic vocalizations (**B**), frequency-modulated 50-kHz ultrasonic vocalizations (**C**), and 22-kHz ultrasonic vocalizations (**D**) emitted in response to tickling. FM = frequency-modulated; S2 = session 2 of the novel object recognition test; USVs = ultrasonic vocalizations. *n* = 40.

Finally, Spearman’s test revealed that the percentages of time spent in novel object exploration during S2 were not significantly correlated with: (i) the overall number of 50-kHz USVs (total calls) (r = 0.08, *p* = 0.63); (ii) the number of flat 50-kHz calls; (r = 0.03, *p* = 0.85); (iii) the number of FM 50-kHz calls (r = 0.10, *p* = 0.53), emitted in the five sessions of tickling (Figure 5A–C). Nevertheless, Spearman’s test revealed that a significant negative correlation existed between the number of 22-kHz USVs emitted in the five sessions of tickling and the percentages of time spent in novel object exploration during S2 (r = −0.36, *p* = 0.02), although the statistical significance of this correlation no longer persisted after Bonferroni’s correction for multiple comparisons (Figure 5D).

### 3.4. Single-Trial Continuous Spontaneous Alternation Behavior Test in a Y Maze and Emission of Ultrasonic Vocalizations

**Figure 6:**
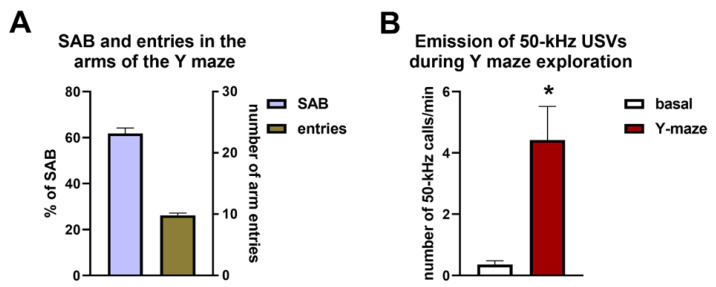
Panel A demonstrates the percentage of spontaneous alternation behavior (left Y axis) and the number of entries in the arms of the Y maze (right Y axis) for all the rats tested. Panel (**B)** demonstrates the emission of 50-kHz ultrasonic vocalizations recorded during Y maze exploration in all the rats tested. * Indicates a significant difference vs. basal. SAB = spontaneous alternation behavior; USVs = ultrasonic vocalizations. *n* = 40 for panel (**A**); *n* = 38 for panel B.

Figure 6A demonstrates the average percentage of SAB and the average number of entries in the arms of the Y maze calculated in all rats. During Y maze exploration, rats emitted a number of 50-kHz USVs per minute that were significantly higher compared with those recorded during the evaluation of basal calling behavior performed before the beginning of tickling (average number of calls = 28.03 ± 6.62 in 8 min of recording, Mann–Whitney U test, U = 276, *p* < 0.01) (Figure 6B), whereas no emission of 22-kHz calls occurred in the same situation. Counterbalancing of NOR and Y maze testing did not affect SAB during Y maze exploration. Indeed, the percentage of SAB was comparable between rats that were first evaluated in the NOR test and then in the Y maze (62.28 ± 2.56) and rats that were first evaluated in the Y maze and then in the NOR test (61.12 ± 3.65).

**Figure 7:**
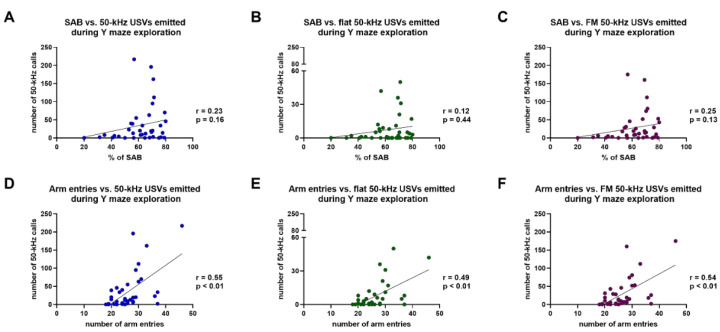
Results of Spearman’s correlation test of the percentages of spontaneous alternation behavior (**A**–**C**) or the number of entries in the arms of the Y maze (**D**–**F**) and the number of total, flat, or frequency-modulated 50-kHz ultrasonic vocalizations emitted during the exploration of the Y maze. FM = frequency-modulated; SAB = spontaneous alternation behavior; USVs = ultrasonic vocalizations. *n* = 38.

Spearman’s test revealed the existence of positive but not significant correlations between the percentages of SAB and: (i) the overall number of 50-kHz USVs (total calls); (ii) the number of flat 50-kHz calls; (iii) the number of FM 50-kHz calls, emitted during Y maze exploration (total calls: r = 0.23, *p* = 0.16; flat calls: r = 0.12, *p* = 0.44; FM calls: r = 0.25, *p* = 0.13) (Figure 7A–C). Conversely, Spearman’s test revealed that significant positive correlations existed between the number of entries in the arms of the Y maze and: (i) the overall number of 50-kHz USVs (total calls); (ii) the number of flat 50-kHz calls; (iii) the number of FM 50-kHz calls, emitted during Y maze exploration (total calls: r = 0.55, *p* < 0.01; flat calls: r = 0.49, *p* < 0.01; FM calls: r = 0.54, *p* < 0.01) (Figure 7D–F).

**Figure 8:**
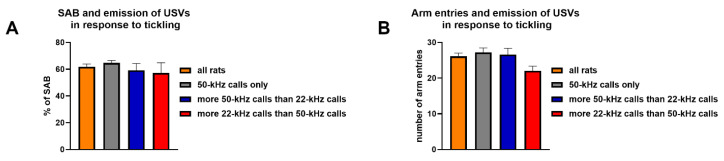
Percentages of spontaneous alternation behavior (**A**) and the number of entries in the arms of the Y maze (**B**) in rats grouped according to the prevalent type of ultrasonic vocalizations (i.e., 22-kHz or 50-kHz calls) emitted in response to tickling. SAB = spontaneous alternation behavior; USVs = ultrasonic vocalizations. *n* = 40 for the groups “all rats”; *n* = 21 for the groups “50-kHz calls only”; *n* = 12 for the groups “more 50-kHz calls than 22-kHz calls”; *n* = 7 for the groups “more 22-kHz calls than 50-kHz calls”.

One-way ANOVA revealed no differences in both the percentages of SAB (F3,76 = 0.68, *p* > 0.05) (Figure 8A) and the number of entries in the arms of the Y maze (F3,76 = 1.49, *p* > 0.05) (Figure 8B) when rats were divided in three groups based on the prevalent type of USVs (i.e., 22-kHz or 50-kHz calls) emitted in response to tickling. Nevertheless, rats that emitted more 22-kHz calls than 50-kHz calls in response to tickling displayed a trend towards a reduction in the number of arm entries. 

**Figure 9:**
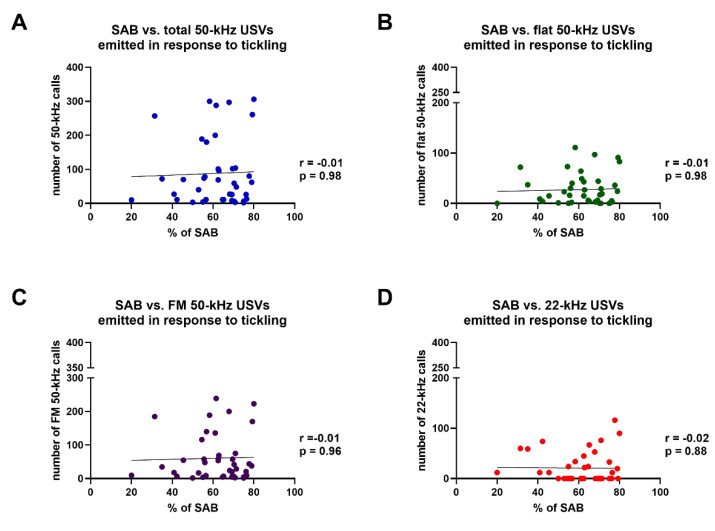
Results of Spearman’s correlation test between the percentages of spontaneous alternation behavior and the numbers of total 50-kHz ultrasonic vocalizations (**A**), flat 50-kHz ultrasonic vocalizations (**B**), frequency modulated 50-kHz ultrasonic vocalizations (**C**) or 22-kHz ultrasonic vocalizations (**D**) emitted in response to tickling. FM = frequency modulated; SAB = spontaneous alternation behavior; USVs = ultrasonic vocalizations. *n* = 40.

Spearman’s test revealed that the percentages of SAB were not significantly correlated with: (i) the overall number of 50-kHz USVs (total calls) (r = −0.005, *p* > 0.05); (ii) the number of flat 50-kHz calls (r = −0.004, *p* > 0.05); (iii) the number of FM 50-kHz calls (r = −0.009, *p* > 0.05); or iv) the number of 22-kHz USVs (r = −0.02, *p* > 0.05) emitted in the five sessions of tickling (Figure 9).

**Figure 10:**
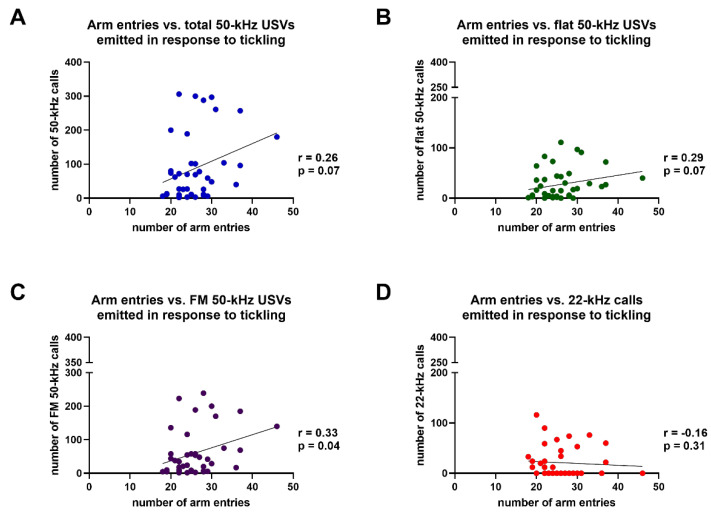
Results of Spearman’s correlation test between the numbers of entries in the arms of the Y maze and the numbers of total 50-kHz ultrasonic vocalizations (**A**), flat 50-kHz ultrasonic vocalizations (**B**), frequency modulated 50-kHz ultrasonic vocalizations (**C**) or 22-kHz ultrasonic vocalizations (**D**) emitted in response to tickling. FM = frequency modulated; USVs = ultrasonic vocalizations. *n* = 40.

Conversely, Spearman’s test revealed that the number of entries in the arms of the Y maze were significantly correlated with the number of FM 50-kHz USVs (r = 0.32, *p* < 0.05) emitted in the five sessions of tickling (Figure 10C), although the statistical significance of this correlation no longer persisted after Bonferroni’s correction for multiple comparisons. Finally, Spearman’s test revealed that the number of entries in the arms of the Y maze were not significantly correlated with the number of: total 50-kHz USVs (r = 0.29, *p* > 0.05), flat 50-kHz calls (r = 0.29, *p* > 0.05), or 22-kHz calls (r = −0.16, *p* > 0.05) emitted in the five sessions of tickling (Figure 10A,B,D). 

## 4. Discussion

In the present study, we measured the emission of 22-kHz and 50-kHz USVs in rats that were subjected to the NOR test and the single-trial continuous SAB test in a Y maze, two experimental paradigms that are used to evaluate memory and do not rely on conditioned stimuli and primary reinforcers. An increased emission of 50-kHz USVs, but not 22-kHz USVs, was observed during the NOR and SAB tests. However, calling behavior during the NOR test was scarce and occurred only in a specific phase of testing. Moreover, the emission of 50-kHz USVs during Y maze exploration was not correlated with the SAB of rats. Nevertheless, differences in object discrimination in the NOR test were observed when rats were grouped according to the prevalent type of USVs emitted in response to tickling, which was performed before the beginning of memory testing.

The NOR test is a behavioral paradigm used to evaluate non-spatial memory in rodents, and we here found that, when considered globally, rats effectively discriminated between novel and old objects, consistent with previous results [21]. However, rats emitted a very low number of 50-kHz USVs during the NOR test and a significant increase in the magnitude of calling behavior was observed in the acquisition phase (S1, when two identical objects were present), but not in the testing phase (S2, when two different objects to be discriminated were present), which indicates that no association existed between object discrimination and modifications in calling behavior. These results indicate that measuring the emission of 50-kHz USVs during testing may be not a useful behavioral marker that reflects the presence of an altered memory function evaluated in experiments of NOR. Nevertheless, additional results obtained in the present study indicate that an interplay may exist between calling behavior and rats’ performance in the NOR test. In fact, differences in object discrimination were observed when rats were grouped according to the prevalent type of USVs (i.e., 22-kHz or 50-kHz calls) emitted in response to tickling that was performed before the beginning of NOR and SAB testing. Tickling is a procedure that may alter the emotional state and that may robustly stimulate calling behavior in rats [12]. Two subpopulations of rats have been characterized that respond differently to tickling in terms of 22-kHz and 50-kHz USV emissions, reflecting the presence of differential dispositional tendency for positive and negative affectivity [30]. In the present study, we replicated these previous findings by showing that the majority of the rats responded to tickling by emitting only or mainly 50-kHz USVs, which may reflect the presence of positive affectivity, and that the remaining minority of rats tested emitted mainly 22-kHz USVs in response to tickling, which may indicate the presence of negative affectivity [30]. Interestingly, the rats that emitted only 50-kHz USVs or that emitted more 50-kHz USVs than 22-kHz USVs in response to tickling effectively discriminated between novel and old objects in the NOR test. Conversely, the rats that emitted more 22-kHz USVs than 50-kHz USVs in response to tickling did not show significant object discrimination in the NOR test.

We may speculate that the abovementioned differences in object discrimination reflect the presence of alterations in memory function rather than in object exploration, since rats spent comparable amounts of time exploring the objects during S1 and S2 of the NOR test, irrespective of the prevalent type of USVs emitted in response to tickling. Moreover, we found that the preference for novel objects in the NOR test displayed a trend towards negative correlation with the emission of 22-kHz USVs in response to tickling, but did not correlate with the overall emission of 50-kHz USVs as well as the emission of FM and flat 50-kHz calls in response to tickling. Taken together, these findings suggest that the emission of 22-kHz USVs in response to tickling may be a behavioral marker potentially predictive of the performance of rats that are subsequently evaluated in the NOR test, and we may propose two explanatory hypotheses in this regard. Based on the previous findings by Burgdorf and coworkers [30], we may speculate that rats that emitted mostly 22-kHz USVs in response to tickling were characterized by a disposition towards negative affectivity, which may have influenced the memory for objects, resulting, in turn, in an impaired performance in the NOR test. This hypothesis would be consistent with previous preclinical studies showing that rats bred for low levels of positive affectivity in response to tickling displayed abnormalities in tests of social behavior and associative learning [30,31], and it would also agree with the results of clinical investigations demonstrating that several abnormalities exist in the cognitive domain of patients suffering from mood disorders [19,20].

Alternatively, we may speculate that rats that emitted mostly 22-kHz USVs in response to tickling were unable to discriminate between objects in the NOR test not because they had a disposition towards negative affectivity, but because they had a dysfunction in specific, yet undefined, brain regions that regulate the emission of 22-kHz USVs as well as item recognition. In this regard, it is worth considering that the emission of 22-kHz USVs can be initiated by the activation of cholinergic transmission at the level of the lateral septum [32,33], and that an impaired object discrimination has been reported in rats that were evaluated in the NOR test after the infusion of pregnenolone in the lateral septum [34]. Hence, we may speculate that an altered function of the lateral septum could be a common mechanism that may explain why rats that emitted mostly 22-kHz USVs in response to tickling also displayed an impaired performance in the NOR test, although the neurochemical events underlying this possible mechanism appear ill defined. In this regard, it is also important to consider that while several cortical and subcortical regions are known to regulate object discrimination in the NOR test [35,36], limited information is available on the brain regions and neurochemical mechanisms that initiate and modulate the emission of 22-kHz USVs. Therefore, clarifying these aspects of the neurobiology of 22-kHz USVs may help to elucidate if an interplay exists between the emission of 22-kHz USVs and unconditioned memories, and how the changes in the emission of these calls relate to the presence of altered memory function evaluated in the NOR test. Furthermore, additional studies will be necessary to clarify whether the emission of 22-kHz USVs may be predictive of and/or associated with the behavioral readouts that are evaluated in other behavioral paradigms that are used to assess non-spatial memory in rats.

The single-trial continuous SAB test in a Y maze is a behavioral paradigm that is used to evaluate non-spatial working memory in rodents, and we here found that rats displayed percentages of SAB in the range of those previously reported [37,38]. Moreover, rats exhibited a significant increase in the emission of 50-kHz USVs during Y maze exploration. Nevertheless, SAB was not significantly correlated with the emission of 50-kHz USVs (total and categorized) recorded during Y maze exploration, although a positive correlation was observed, nor was SAB correlated with the emission of 50-kHz USVs (total and categorized) in response to tickling. These results indicate that the emission of 50-kHz USVs is not a behavioral marker that may be associated with or predict the SAB of rats tested in a Y maze.

On the other hand, positive and significant correlations were found between the entries in the arms of the Y maze and the emission of 50-kHz USVs (total and categorized) recorded either during Y maze exploration or in response to tickling performed before memory testing. The number of arm entries in tests of continuous SAB in a Y maze can provide a measure of locomotor activity [22], which could suggest that an interplay exists between the emission of 50-kHz USVs and locomotion in rats during Y maze testing. However, several lines of evidence indicate that the emission of 50-kHz USVs cannot be simply considered a byproduct of locomotion, but it rather reflects the presence of arousal/positive affect [39,40]. On these bases, one hypothesis that could explain the increased emission of 50-kHz USVs during Y maze exploration is that this situation elicited arousal/positive affect in rats and, accordingly, calling behavior. A possible mechanism that could underlie the emission of 50-kHz USVs during Y maze exploration is the curiosity towards a novel environment. Indeed, curiosity may be associated with increased arousal [41], and curiosity is thought to be a factor that drives the exploratory behavior of rats exposed to a Y maze [22]. Moreover, the existence of an interplay between curiosity and emission of USVs has been proposed by studies that evaluated calling behavior during social contacts in mice [6,42]. Nevertheless, it is noteworthy that rats may also emit 50-kHz USVs in situations that are not necessarily pleasurable or appetitive for them [43]. Hence, an alternative hypothesis may be that the emission of 50-kHz USVs during Y maze exploration did not stem from changes in the affective state of rats but from other, yet undefined, mechanisms. Furthermore, it is noteworthy that we found a trend to negative correlation between the entries in the arms of the Y maze and the emission of 22-kHz USVs recorded in response to tickling. Considering all the findings, further investigations are warranted to clarify whether the interplay observed here between calling behavior and entries in the arm of a Y maze has behavioral significance, and whether it may have any relevance as a behavioral marker of spatial memory. Indeed, it has to be remarked that in the continuous SAB test in a Y maze, it is the sequence of arm entries, rather than the number of arm entries, that is used as a behavioral readout to assess spatial working memory [22]. 

Earlier investigations by us and others have demonstrated an increased emission of 50-kHz USVs in rats upon the presentation of environmental stimuli that were previously paired with either a social reward or the administration of drugs that possess rewarding properties [9,10,11,12,13,14,15,16,17,44,45]. Moreover, a very recent study has demonstrated a persistently decreased emission of FM 50-kHz USVs in rats subjected to fear conditioning [18]. Based on these findings, the changes in the emission of 50-kHz USVs may be regarded as a behavioral marker of conditioned memories, and it has been suggested that such calling behavior may capture the affective component of conditioned memories [18]. In the present study, we obtained evidence to suggest that measuring the emission of USVs may deserve further consideration as a potential behavioral marker also in studies of memory based on the use of paradigms that do not rely on conditioned stimuli/primary reinforcers. More specifically, according to the presented data, we propose that the emission of 50-kHz USVs may be not a useful behavioral marker associated with, or predictive of, object discrimination in rats tested in the NOR and of SAB in rats tested in a Y maze. However, the results of this study suggest that an interplay may exist between the emission of 22-kHz USVs and object discrimination in the NOR test, although a more detailed investigation of the relationship between the emission of 22-kHz USVs and unconditioned non-spatial memory is needed. 

The present study may have a potential limitation in that it did not include a pure control group of rats that were not subjected to tickling. Our results indicate that prior tickling experience did not affect memory performance of rats in the NOR test, at least in those animals that emitted only or mostly 50-kHz USVs, which were found to effectively discriminate between novel and old objects. Moreover, prior tickling experience did not affect the pattern of Y maze exploration, since rats displayed percentages of SAB that were in the range of those previously described. Nevertheless, we cannot rule out the possibility that prior tickling experience may have elicited an enduring influence on rats’ vocal behavior, which eventually resulted in a dissimilar emission of USVs between the NOR and SAB tests. In this regard, it is also noteworthy that the lack of USV emissions observed here during the NOR test may appear unexpected, and in contrast to the increased calling behavior recorded during Y maze exploration. In fact, previous studies have demonstrated that the exposure to novel environments and situations of novelty, as may be the case for the presentation of objects during the NOR test, may stimulate the emission of USVs in rats [43,46,47]. In this connection, we cannot exclude the possibility that methodological issues related to test implementation contributed, at least in part, to the differences in calling behavior observed here between the NOR and SAB tests. Indeed, during the NOR test, rats were not in direct contact with sawdust, since the bottom of the test cage was covered with an elevated grid that had a handful of sawdust below it. Importantly, it has been demonstrated that contact with sawdust is a factor that facilitates calling behavior in rats [47]. Conversely, the Y maze used in this study had the bottom covered with sawdust and also had the walls painted black: these factors may have resulted in a more favorable environment for rats, and in turn could have facilitated the emission of USVs. Based on these considerations, we suggest that methodological issues are carefully examined in future studies, since doing so will help to elucidate the behavioral significance of the USVs that are recorded in rats subjected to tests of memory.

## 5. Conclusions

Elucidating the networking between the emission of USVs and memory function in rats appears of interest in the consideration of the evidence, suggesting that a relationship may exist between changes in the affective state and alterations in memory function [19,20], and in light of the evidence that the emission of USVs is a behavioral marker of affect in rats [39,40]. Accordingly, further clarification of how the emission of USVs varies in magnitude and type (i.e., 22-kHz vs. 50-kHz calls) in experimental paradigms that evaluate different forms of memory (i.e., non-spatial vs. spatial, long-term vs. short term) is of interest, as it may potentially contribute to increase the amount of information that can be collected in studies of memory in rats. 

## Figures and Tables

**Table 1:** Cumulative times of object exploration during sessions 1 and 2 of the novel object recognition test in rats grouped according to the prevalent type of ultrasonic vocalizations emitted in response to tickling. Exploration times are reported as the average number of seconds ± S.E.M. S1 = session 1; S2 = session 2.

	Cumulative Time of Object Exploration during S1	Cumulative Time of Object Exploration during S2
all rats	13.59 ± 1.29	15.33 ± 1.21
50-kHz calls only	13.21 ± 1.43	14.99 ± 1.38
more 50-kHz calls than 22-kHz calls	12.27 ± 1.96	14.75 ± 2.26
more 22-kHz calls than 50-kHz calls	16.99 ± 5.15	17.34 ± 4.19

## Data Availability

Data will be made available by the corresponding author upon reasonable request.
